# Perceived Knowledge and Confidence of Beta-Lactam Allergy Management Among Pharmacy Students on Advanced Pharmacy Practice Experiences

**DOI:** 10.3390/pharmacy13050135

**Published:** 2025-09-18

**Authors:** Jamie L. Wagner, David R. Oliver, Bruce M. Jones, Kayla R. Stover, Misha T. Watts, Wesley D. Kufel, Lena McDowell, Edoabasi U. McGee, T. Lynn Stevenson, Christopher M. Bland

**Affiliations:** 1 Mount Auburn Hospital, Cambridge, MA 02138, USA; jamie.leigh.wagner@gmail.com; 2 Archbold Medical Center, Thomasville, GA 31792, USA; ryano24601@gmail.com; 3 St. Joseph’s/Candler Health System, Inc., Savannah, GA 31405, USA; brucemjones@hotmail.com; 4 Department of Pharmacy Practice, University of Mississippi School of Pharmacy, Jackson, MS 39216, USA; kstover@umc.edu; 5 Division of Experience Programs, University of Georgia College of Pharmacy, Savannah, GA 31405, USA; mthomaso2@uga.edu; 6 Department of Pharmacy Practice, Binghamton University School of Pharmacy and Pharmaceutical Sciences, Binghamton, NY 13902, USA; wkufel@binghamton.edu; 7 Department of Pharmacy Practice, Auburn University Harrison School of Pharmacy, Auburn, AL 36849, USA; mcdowld@auburn.edu (L.M.); tls0002@auburn.edu (T.L.S.); 8 Department of Pharmacy Practice, Philadelphia College of Osteopathic Medicine School of Pharmacy, Suwanee, GA 30024, USA; edoabasimc@pcom.edu

**Keywords:** beta-lactams, curriculum, drug hypersensitivity, penicillins

## Abstract

Pharmacist engagement in allergy clarification has demonstrated increased appropriate antibiotic use. The purpose of this study was to determine the knowledge and confidence of pharmacy students in their final professional year regarding beta-lactam (BL) allergies. Students from 5 schools of pharmacy participated in a 22-question survey pertaining to experience with drug allergies, knowledge of BL allergies, and confidence regarding BL allergy management. Data were summarized among all respondents and further analyzed by infectious disease (ID) interest. A total of 160/521 students responded to the survey (31%). Most students (73%) had no course dedicated to drug allergies; however, 84% indicated the topic was taught within the curriculum. Students with an ID interest had a higher perceived knowledge regarding the details of penicillin skin testing (62% vs. 32%), clinical implications of penicillin skin test results (87% vs. 70%), and the principles behind a graded and direct penicillin challenge (64% vs. 41%). These students were more confident in educating patients about a perceived penicillin allergy (34% vs. 15%). Perceived knowledge and confidence of BL allergies were low, especially in high-level interventions. Targeted training in beta-lactam allergy recognition and management within the curriculum should be considered to improve upon these findings.

## 1. Introduction

**Figure 1:**
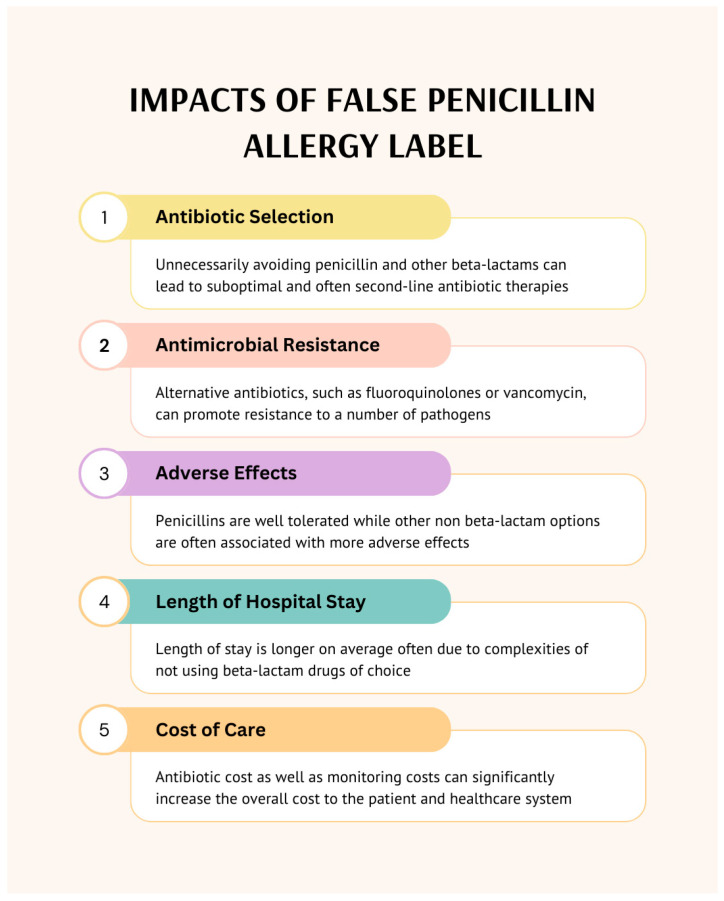
Impacts of False Penicillin Allergy Label.

With up to 12% of the United States population reporting a penicillin allergy and 90% of patients with a documented penicillin allergy retaining the ability to tolerate a beta-lactam (BL), the importance of clarifying the penicillin allergy is imperative for optimizing antimicrobial treatment, decreasing adverse effects and antimicrobial resistance, and decreasing costs (Figure 1) [1,2,3,4]. While allergy testing is typically handled by allergy specialists, recent literature supports the use of pharmacists to help clarify and de-label penicillin allergies, often as leaders of penicillin allergy assessment and intervention programs [5,6,7]. In these studies, pharmacists were the leaders of education, guideline implementation, and allergy assessment that improved not only allergy documentation, but also the appropriate use of BL antibiotics in hospitalized patients with a reported BL allergy. Additionally, allergy assessment and management falls under all the elements within comprehensive medication management (CMM), making pharmacists the ideal provider to engage in this care.

In the United States, CMM, as well as collaborative practice agreements (CPAs), allow pharmacists to take ownership in reviewing medication therapy, contributing to medication records, developing medication-related action plans, intervening and/or providing referrals, and documenting and following-up with patients [8]. Through a CPA or with provider status in several states within the US, pharmacists can complete a thorough medication allergy history, perform medication skin testing (e.g., penicillin), interpret skin test results, and utilize cross-reactivity data to manage medications to optimize adherence, improve outcomes, and reduce healthcare costs [8]. One publication detailing procedures for performing penicillin skin testing demonstrated that a significant number of state boards of pharmacy, when contacted, noted pharmacists were either not allowed to perform testing or had no information, which could be a significant barrier when providing this service [9]. However, a number of important BL allergy interventions can be completed to aid in management (e.g., updating the medical record with details of the allergic event or confirming the allergy was actually a side effect).

Even with the importance of appropriate allergy assessment and management, a 2017 survey of 276 attending physicians, residents, advanced practice practitioners, and pharmacists practicing in internal medicine (general and subspecialty), surgery (general and subspecialty), and obstetrics and gynecology demonstrated limited knowledge and understanding of BL allergy assessment and intervention [10]. However, despite only comprising 12% of the included participants, pharmacists were more knowledgeable regarding management of BL allergic patients, specifically regarding knowledge that BL allergies can resolve with time and that 90% of BL-allergic patients will tolerate another BL. Pharmacists were also more likely to review the patient’s medical record for medication administration to determine past exposure to BLs.

While pharmacy students are taught to assess allergies as part of the Pharmacists’ Patient Care Process, including the collect, assess, and plan steps, a specific focus on how to clarify or manage patients with medication allergies may not be emphasized in the didactic curriculum [11]. A recent multicenter national survey of infectious disease (ID) faculty at pharmacy schools within the U.S. demonstrated significant variability within pharmacy school curricula and most faculty perceived students to be unprepared to evaluate and manage beta-lactam allergies [12]. Additionally, pharmacy students are continually assessed for Advanced Pharmacy Practice Experience (APPE) readiness through their overall grade point average, comprehensive assessments, simulated patient encounters, objective structured clinical exams (OSCEs), and introductory pharmacy practice experiences (IPPEs) [13]. However, students will likely gain significant exposure to patients in need of allergy clarification and management on their APPEs in their final year of pharmacy school, where they spend 40 h a week in different patient care settings. Combining the increasing autonomy of pharmacist-driven patient care with the importance of optimizing medication use, this study seeks to determine the perceived readiness of APPE students regarding BL allergies and allergy management.

## 2. Materials and Methods

### 2.1. Study Design and Population

This was a cross-sectional, multicenter, anonymous, electronic survey distributed to pharmacy students on APPEs. A total of 521 pharmacy students (all students on APPEs were eligible to participate with no exclusions) from 5 schools of pharmacy (mix of public and private) in the United States were eligible to participate in this study. These schools were located in Alabama, Georgia, Mississippi, and New York. Survey responses that were either incomplete or missing data were excluded. This study was deemed exempt by the institutional review board at all participating schools of pharmacy due to not involving human clinical subjects (PROJECT00000892).

### 2.2. Survey Instrument

A 22-item survey instrument was developed to collect data about APPE pharmacy students’ perceived knowledge and confidence regarding BL allergies (Table 1). This survey was created using Qualtrics survey software (Qualtrics, Inc., Provo, UT, USA) and developed based on expert opinions of ID pharmacists with experience in BL allergy management (authors Jones and Bland). The instrument was divided into three primary areas: Introduction, Self-Perceived Knowledge, and Level of Confidence. The Introduction to the survey assessed student’s didactic and curriculum exposure to BL allergies and their management using 7 questions. Self-Perceived Knowledge was assessed using a modified Likert scale of 4 categories, ranging from “not knowledgeable” to “very knowledgeable,” to assess students’ self-perceived knowledge of various BL allergy topics in 9 questions. Level of Confidence (self-perceived) assessed students’ confidence in applying their knowledge to various BL management scenarios using 6 questions. Questions included curriculum exposure to drug allergies, including management of allergies, exposure to ID rotations, knowledge of the types, signs, symptoms, assessment, management, etc., of allergic reactions, and confidence questions regarding patient education, penicillin skin testing, and clinical therapy in patients with BL allergies. Self-Perceived Level of Confidence was also assessed using a modified Likert scale of 4 categories, ranging from “not confident at all” to “high confidence applying independently.” The survey was piloted through several authors to improve validity, readability, and logistical integrity prior to distribution.

### 2.3. Survey Administration

Contact information for each pharmacy student was obtained from the investigator affiliated with each school of pharmacy. Surveys were distributed electronically via email to pharmacy students between August 2019 and February 2020. Each electronic survey was sent with an invitation letter that included appropriate informed consent information. Participation was voluntary, and by entering, respondents agreed to survey participation. The survey was anonymous, and all survey responses were de-identified. All survey responses were collected and stored in Qualtrics survey software. Students were sent at least 2 reminder emails to complete the survey.

### 2.4. Statistical Analyses

Data analysis was performed using SPSS Statistics, version 26.0 (IBM, Chicago, IL, USA). Descriptive statistics were used to summarize collected data. Student responses were further categorized into two groups: those with an interest in ID (e.g., scheduled or requested ID APPE) and those without an interest in ID (e.g., no scheduled or requested ID APPE). Categorical data were analyzed using Chi-Square or Fischer’s Exact test, as appropriate. All statistical tests were 2-tailed with a *p* value < 0.05 indicating statistical significance.

## 3. Results

All 521 eligible APPE students were emailed, and 160 students participated in the study, resulting in an overall response rate of 31%. There were 61 (38%) students who had expressed interest in ID, with 9 (15%) students already exposed to an ID rotation at the time of this survey, while 99 (62%) students did not express an interest in ID. When asked whether the school of pharmacy had a class or course (e.g., at least 1 credit hour) dedicated to drug allergies, 116 (73%) students responded no, with significantly more students in the non-ID group stating they did not have a class/course than the ID group (81% vs. 59%; *p* = 0.003). However, 135 (84%) students indicated that management of BL allergies was taught within the curriculum as a single lecture or series of lectures within a class/course, with no difference observed between groups.

**Figure 2:**
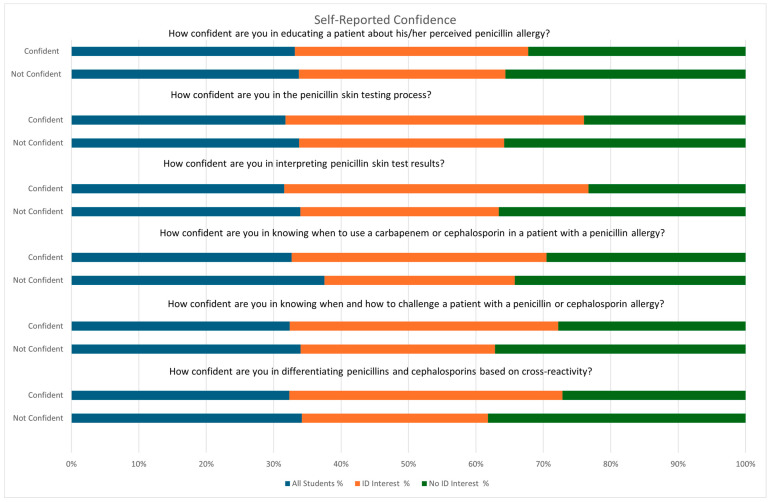
Summary of Self-Reported Confidence.

Overall knowledge responses can be found in Table 2. Most students reported feeling either somewhat knowledgeable (n = 68; 43%) or knowledgeable (n = 73; 46%) about the 4 types of allergic hypersensitivity reactions; 63 (40%) students reported feeling knowledgeable, and 84 (53%) felt they were very knowledgeable on being able to recognize the signs and symptoms of anaphylaxis. The most common response for penicillin skin test knowledge questions was not feeling knowledgeable or feeling somewhat knowledgeable. Half of respondents felt they were not knowledgeable in understanding principles of direct and graded penicillin challenge. Significantly more students in the ID group perceived themselves as knowledgeable (n = 24; 39%) or very knowledgeable (n = 14; 23%) about the details surrounding penicillin skin testing compared to 24% (n = 24; *p* = 0.043) and 8% (n = 8; *p* = 0.008), respectively, in the non-ID group. When asked about the principles of a graded and direct penicillin challenge, as well as the clinical implications of penicillin skin test results, more students in the non-ID group felt they were not knowledgeable (penicillin challenge: 59% vs. 36%; *p* = 0.006; clinical implications: 30% vs. 13%; *p* = 0.013). There were no differences between groups for perceived understanding when penicillin skin testing is not indicated and knowledge on how to counsel and educate patients on their penicillin allergy. Overall perceived confidence among survey respondents for most questions was either not confident at all or just basic understanding. Finally, students in the ID group felt more confident than the non-ID group in the following categories: educating a patient about a perceived penicillin allergy (34% vs. 15%; *p* = 0.005), understanding the penicillin skin testing process (13% vs. 3%; *p* = 0.022), and interpreting penicillin skin test results (25% vs. 11%; *p* = 0.022) (Table 3). Figure 2 provides a visual summary of student self-reported confidence. “Not confident at all” and “basic understanding, but not confident” were placed into the “Not Confident” group while “confident with minimal supervision” and “highly confident to apply independently” were placed into the “Confident” group.

## 4. Discussion

Avoidance of BLs due to a perceived penicillin allergy can result in increased risk of adverse events, antibiotic-related costs, hospital length of stay, and a higher incidence of drug-resistant, hospital-acquired infections [14,15,16]. Pharmacists are well-qualified and positioned to provide comprehensive BL allergy assessment and intervention due to their expertise and training [17]. The ubiquitous nature of pharmacists, combined with approximately 90% of the United States population living within 5 miles of a pharmacy, allows for a great opportunity to provide these valuable services in both inpatient and ambulatory settings [18]. Therefore, proper education and training of pharmacists within these interventions would provide a significant opportunity and benefit for patients and overall public health to ultimately de-label patients of their allergy [14,15,16]. Additionally, with the expansion of The Joint Commission’s requirement for antimicrobial stewardship in the outpatient setting, ambulatory care pharmacists are in the ideal position to expand BL allergy assessment through penicillin skin testing, direct oral challenges, or referrals to allergy specialists [19].

The findings from our study across multiple schools of pharmacy suggested that while pharmacy students on APPE rotations receive instruction at some point in the curriculum, a more focused approach should be considered to properly train students on this ubiquitous clinical conundrum within practice. Learning experiences should be practical, case-based, work-integrated, and should support development of skills to work professionally based on knowledge within the discipline of BL allergies, specifically penicillin allergies [12]. While students overall felt they were knowledgeable and confident with lower-level BL allergy statements, such as understanding basics of allergic reactions, our survey demonstrated significant gaps in perceived knowledge and confidence with BL allergy intervention strategies, such as direct or graded challenge, which can often be used to de-label a patient’s allergy with minimal resources. Students with an interest in ID had greater perceived knowledge and confidence within a number of higher-level intervention strategy domains, such as penicillin skin testing. While few data have assessed comprehensive strategies for BL allergy education, a recent quasi-experimental study by Kufel et al. across two schools of pharmacy demonstrated increased knowledge scores in relation to penicillin allergy detriments, cross-reactivity with other BLs, and understanding a correct stepwise approach to skin testing. Confidence scores also increased significantly with regard to penicillin skin testing [20]. These objectives were achieved through a brief lecture on penicillin allergy assessment and skin testing as well as a lab-based simulation that allowed students to perform a portion of the penicillin skin test, participate in penicillin allergy interview sessions, and reconcile appropriate management strategies for penicillin allergy case vignettes.

Students who were interested in ID felt they were significantly more knowledgeable and confident in higher-level interventions, such as graded challenge and penicillin skin testing. The increase in knowledge and confidence might be due to the ID interest of the students; however, pharmacists outside of ID are also becoming more involved with penicillin skin testing, as it is an important tool in the assessment and subsequent de-labeling of patients who report a Type I penicillin allergy, such as hives or anaphylaxis [9]. A study evaluating cost savings associated with interventions performed by APPE students noted that one of the top 3 intervention types resulting in the highest amount of money saved per intervention was identifying potential allergic reactions ($1090 USD per intervention) [21].

This study is not without limitations. First, we had a lower survey response rate than expected despite keeping the survey window open for an extended period of time, possibly leading to selection bias of responding students. However, low response rates may not actually induce nonresponse bias and waiting for a higher response rate may induce other measurement problems [22]. Additionally, the students surveyed were from 5 schools of pharmacy and may not represent the curriculum experienced by all APPE pharmacy students. Lastly, by surveying APPE students, we intended to direct our perceived knowledge and confidence questions specifically towards what was learned in the didactic curriculum; however, exposure on APPE rotations could have influenced the responding students. While all schools asked for rotation preferences, it is possible that students assigned an ID rotation was due to availability and not interest. Schools participating in this study ask students for preferences and while these are most often met for students, sometimes students may have been placed within an infectious diseases APPE due to limited availability of other APPEs. Future studies could survey students post graduation to definitively know who completed an ID rotation, but limited participation is likely due to students no longer attending the college or school of pharmacy and are preparing for licensure exams and job placement. Finally, students were not all surveyed in the same APPE rotation block, so overall experience at the time of the survey was not exact.

## 5. Conclusions

Many students reported exposure to the management of BL allergies during their didactic curriculum, but this did not translate into increased perceived knowledge and confidence across a number of low-level and high-level interventions when in their final professional year. Students who had an interest in ID conveyed greater perceived knowledge and confidence when compared to students without an interest in ID. This included a statistically significant higher confidence of approximately 20% in educating patients about their penicillin allergy. Strong consideration for integrating comprehensive beta-lactam allergy didactic material should be considered to increase knowledge and confidence across the continuum of beta-lactam allergy assessment and intervention with the goal of ultimately de-labeling the patient’s medical record once licensed for practice. In addition to continued improvement of the didactic curriculum within penicillin allergy assessment and intervention, enhanced education and training of licensed pharmacists, specifically within the management of beta-lactam allergies, can potentially not only improve patient care across a broad number of clinical settings, but also improve skills of preceptors on APPEs to enhance student learning long-term and encourage de-labeling of penicillin allergies.

## Figures and Tables

**Table 1:** Beta-Lactam Survey Questions.

Introduction Questions (Yes or No)
Would you like to participate in the above described study?
Did your school of pharmacy have a class or course dedicated to drug allergies as part of its curriculum?
Did your school dedicate a lecture within a course to drug allergies?
Did any of your courses teach management of beta-lactam allergies as part of its curriculum?
Was management of beta-lactam allergies taught at all in any of your courses?
Have you completed or do you have an infectious diseases rotation scheduled during your APPE year?
Did you request an infectious diseases rotation?
**Self-Perceived Knowledge Questions rated as: 1 (Not Knowledgeable), 3 (Somewhat Knowledgeable), 5 (Knowledgeable), or 7 (Very Knowledgeable)**
I know the four types of allergic hypersensitivity reactions.
I know and can recognize the signs and symptoms of anaphylaxis.
I understand how to counsel and educate patients on their penicillin allergy.
I know what penicillin skin testing entails.
I know when penicillin skin testing is not indicated.
I know the clinical implications of penicillin skin test results.
I understand when desensitization would be appropriate for patient care.
I understand the principles of graded penicillin challenge and direct penicillin challenge.
I understand cross-reactivity between penicillins and cephalosporins.
**Level of Confidence Questions rated as: Not confident at all, Basic Understanding, but not confident, Confident with minimal supervision, or Highly Confident to Apply Independently**
How confident are you in educating a patient about his/her perceived penicillin allergy?
How confident are you in the penicillin skin testing process?
How confident are you in interpreting penicillin skin test results?
How confident are you in knowing when to use a carbapenem or cephalosporin in a patient with a penicillin allergy?
How confident are you in knowing when and how to challenge a patient with a penicillin or cephalosporin allergy?
How confident are you in differentiating penicillins and cephalosporins based on cross-reactivity?

**Table 2:** Student perceived knowledge pertaining to drug allergies and penicillin allergy management (N = 160).

	All Students n (%)	ID Interest n (%)	No ID Interest n (%)	*p* Value
I know the four types of allergic hypersensitivity reactions.				
Not knowledgeable	3 (1.9)	1 (1.6)	2 (2)	1.000
Somewhat knowledgeable	68 (42.5)	25 (41)	43 (43.4)	0.761
Knowledgeable	73 (45.6)	30 (49.2)	43 (43.4)	0.478
Very knowledgeable	16 (10)	5 (8.2)	11 (11.1)	0.551
I know and can recognize the signs and symptoms of anaphylaxis. (N = 159; ID rotation missing N = 1)				
Not knowledgeable	1 (0.6)	1 (1.7)	0 (0)	0.377
Somewhat knowledgeable	11 (6.9)	5 (8.3)	6 (6.1)	0.749
Knowledgeable	63 (39.6)	26 (43.3)	37 (37.4)	0.456
Very knowledgeable	84 (52.8)	28 (46.7)	56 (56.6)	0.226
I understand how to counsel and educate patients on their penicillin allergy.				
Not knowledgeable	3 (1.9)	1 (1.6)	2 (2)	1.000
Somewhat knowledgeable	40 (25)	16 (26.2)	24 (24.2)	0.778
Knowledgeable	70 (43.8)	23 (37.7)	47 (47.5)	0.226
Very knowledgeable	47 (29.4)	21 (34.4)	26 (26.3)	0.271
I know what penicillin skin testing entails.				
Not knowledgeable	37 (23.1)	10 (16.4)	27 (27.3)	0.113
Somewhat knowledgeable	53 (33.1)	13 (21.3)	40 (40.4)	0.013 *
Knowledgeable	48 (30)	24 (39.3)	24 (24.2)	0.043 *
Very knowledgeable	22 (13.8)	14 (23)	8 (8.1)	0.008 *
I know when penicillin skin testing is not indicated.				
Not knowledgeable	60 (37.5)	18 (29.5)	42 (42.4)	0.101
Somewhat knowledgeable	56 (35)	21 (34.4)	35 (35.4)	0.905
Knowledgeable	26 (16.3)	13 (21.3)	13 (13.1)	0.173
Very knowledgeable	18 (11.3)	9 (14.8)	9 (9.1)	0.271
I know the clinical implications of penicillin skin test results.				
Not knowledgeable	38 (23.8)	8 (13.1)	30 (30.3)	0.013 *
Somewhat knowledgeable	62 (38.8)	24 (39.3)	38 (38.4)	0.904
Knowledgeable	41 (25.6)	20 (32.8)	21 (21.2)	0.103
Very knowledgeable	19 (11.9)	9 (14.8)	10 (10.1)	0.377
I understand when desensitization would be appropriate for patient care.				
Not knowledgeable	29 (18.1)	11 (18)	18 (18.2)	0.981
Somewhat knowledgeable	65 (40.6)	23 (37.7)	42 (42.4)	0.555
Knowledgeable	41 (25.6)	13 (21.3)	28 (28.3)	0.327
Very knowledgeable	25 (15.6)	14 (23)	11 (11.1)	0.045 *
I understand the principles of graded penicillin challenge and direct penicillin challenge.				
Not knowledgeable	80 (50)	22 (36.1)	58 (58.6)	0.006 *
Somewhat knowledgeable	38 (23.8)	20 (32.8)	18 (18.2)	0.035 *
Knowledgeable	22 (13.8)	11 (18)	11 (11.1)	0.217
Very knowledgeable	20 (12.5)	8 (13.1)	12 (12.1)	0.854
I understand cross-reactivity between penicillins and cephalosporins.				
Not knowledgeable	3 (1.9)	1 (1.6)	2 (2)	1.000
Somewhat knowledgeable	38 (23.9)	14 (23)	24 (24.5)	0.825
Knowledgeable	79 (49.7)	30 (49.2)	49 (50)	0.920
Very knowledgeable	39 (24.5)	16 (26.2)	23 (23.5)	0.694

ID = infectious disease; * Statistically significant.

**Table 3:** Student perceived confidence pertaining to penicillin allergy management (N = 160).

Questions	All Students n (%)	ID Interest n (%)	No ID Interest n (%)	*p* Value
How confident are you in educating a patient about his/her perceived penicillin allergy?				
Not confident at all	3 (1.9)	2 (3.3)	1 (1)	0.558
Basic understanding, but not confident	49 (30.6)	16 (26.2)	33 (33.3)	0.344
Confident with minimal supervision	72 (45)	22 (36.1)	50 (50.5)	0.075
Highly confident to apply independently	36 (22.5)	21 (34.4)	15 (15.2)	0.005 *
How confident are you in the penicillin skin testing process?				
Not confident at all	70 (43.8)	21 (34.4)	49 (49.5)	0.062
Basic understanding, but not confident	58 (36.3)	23 (37.7)	35 (35.4)	0.764
Confident with minimal supervision	21 (13.1)	9 (14.8)	12 (12.1)	0.632
Highly confident to apply independently	11 (6.9)	8 (13.1)	3 (3)	0.022 *
How confident are you in interpreting penicillin skin test results?				
Not confident at all	68 (42.8)	20 (33.3)	48 (48.5)	0.061
Basic understanding, but not confident	54 (34)	20 (33.3)	34 (34.3)	0.896
Confident with minimal supervision	26 (16.4)	15 (25)	11 (11.1)	0.022 *
Highly confident to apply independently	11 (6.9)	5 (8.3)	6 (6.1)	0.749
How confident are you in knowing when to use a carbapenem or cephalosporin in a patient with a penicillin allergy?				
Not confident at all	15 (19.4)	6 (9.8)	9 (9.1)	0.875
Basic understanding, but not confident	77 (48.1)	25 (41)	52 (52.5)	0.156
Confident with minimal supervision	48 (30)	22 (36.1)	26 (26.3)	0.189
Highly confident to apply independently	20 (12.5)	8 (13.1)	12 (12.1)	0.854
How confident are you in knowing when and how to challenge a patient with a penicillin or cephalosporin allergy?				
Not confident at all	37 (23.1)	14 (23)	23 (23.2)	0.967
Basic understanding, but not confident	59 (36.9)	17 (27.9)	42 (42.4)	0.064
Confident with minimal supervision	44 (27.5)	21 (34.4)	23 (23.2)	0.124
Highly confident to apply independently	20 (12.5)	9 (14.8)	11 (11.1)	0.499
How confident are you in differentiating penicillins and cephalosporins based on cross-reactivity?				
Not confident at all	16 (10)	4 (6.6)	12 (12.1)	0.255
Basic understanding, but not confident	75 (46.9)	24 (39.3)	51 (51.5)	0.134
Confident with minimal supervision	42 (26.3)	20 (32.8)	22 (22.2)	0.140
Highly confident to apply independently	27 (16.9)	13 (21.3)	14 (14.1)	0.240

ID = infectious disease; * Statistically significant.

## Data Availability

The data presented in this study are available upon request from the corresponding author.
